# Towards Precision Medicine in Gestational Diabetes: Pathophysiology and Glycemic Patterns in Pregnant Women With Obesity

**DOI:** 10.1210/clinem/dgad168

**Published:** 2023-03-23

**Authors:** Sara L White, Albert Koulman, Susan E Ozanne, Samuel Furse, Lucilla Poston, Claire L Meek

**Affiliations:** Department of Women and Children’s Health, School of Life Course and Population Sciences, Faculty of Life Sciences and Medicine, King’s College London, London, SE1 7EH, UK; Core Metabolomics and Lipidomics Laboratory, Wellcome Trust-MRC Institute of Metabolic Science, University of Cambridge, Addenbrooke’s Treatment Centre, Cambridge, CB2 0QQ, UK; Wellcome Trust-MRC Institute of Metabolic Science, University of Cambridge, Addenbrooke’s Treatment Centre, Cambridge, CB2 0QQ, UK; Wellcome Trust-MRC Institute of Metabolic Science, University of Cambridge, Addenbrooke’s Treatment Centre, Cambridge, CB2 0QQ, UK; Core Metabolomics and Lipidomics Laboratory, Wellcome Trust-MRC Institute of Metabolic Science, University of Cambridge, Addenbrooke’s Treatment Centre, Cambridge, CB2 0QQ, UK; Wellcome Trust-MRC Institute of Metabolic Science, University of Cambridge, Addenbrooke’s Treatment Centre, Cambridge, CB2 0QQ, UK; Department of Women and Children’s Health, School of Life Course and Population Sciences, Faculty of Life Sciences and Medicine, King’s College London, London, SE1 7EH, UK; Core Metabolomics and Lipidomics Laboratory, Wellcome Trust-MRC Institute of Metabolic Science, University of Cambridge, Addenbrooke’s Treatment Centre, Cambridge, CB2 0QQ, UK; Wellcome Trust-MRC Institute of Metabolic Science, University of Cambridge, Addenbrooke’s Treatment Centre, Cambridge, CB2 0QQ, UK; Department of Clinical Biochemistry/Wolfson Diabetes & Endocrine Clinic, Cambridge University Hospitals NHS Foundation Trust, Cambridge, CB2 0QQ, UK

**Keywords:** glycemia, lipidomics, triglycerides, de novo lipogenesis, oral glucose tolerance test, pregnancy, gestational diabetes, pathophysiology, insulin resistance, hyperglycemia, fasting hyperglycemia, lipid dysfunction, precision medicine, insulin resistance, obesity, oral glucose tolerance test, pregnancy, subgroups

## Abstract

**Aims:**

Precision medicine has revolutionized our understanding of type 1 diabetes and neonatal diabetes but has yet to improve insight into gestational diabetes mellitus (GDM), the most common obstetric complication and strongly linked to obesity. Here we explored if patterns of glycaemia (fasting, 1 hour, 2 hours) during the antenatal oral glucose tolerance test (OGTT), reflect distinct pathophysiological subtypes of GDM as defined by insulin secretion/sensitivity or lipid profiles.

**Methods:**

867 pregnant women with obesity (body mass index ≥ 30 kg/m^2^) from the UPBEAT trial (ISRCTN 89971375) were assessed for GDM at 28 weeks’ gestation (75 g oral glucose tolerance test OGTT; World Health Organization criteria). Lipid profiling of the fasting plasma OGTT sample was undertaken using direct infusion mass spectrometry and analyzed by logistic/linear regression, with and without adjustment for confounders. Insulin secretion and sensitivity were characterized by homeostatic model assessment 2b and 2s, respectively.

**Results:**

In women who developed GDM (n = 241), patterns of glycaemia were associated with distinct clinical and biochemical characteristics and changes to lipid abundance in the circulation. Severity of glucose derangement, rather than pattern of postload glycaemia, was most strongly related to insulin action and lipid abundance/profile. Unexpectedly, women with isolated postload hyperglycemia had comparable insulin secretion and sensitivity to euglycemic women, potentially indicative of a novel mechanistic pathway.

**Conclusions:**

Patterns of glycemia during the OGTT may contribute to a precision approach to GDM as assessed by differences in insulin resistance/secretion. Further research is indicated to determine if isolated postload hyperglycemia reflects a different mechanistic pathway for targeted management.

Diabetes is now widely recognized to result from a variety of pathophysiological processes, facilitating a precision medicine approach to disease progression, treatment, and outcomes (1). Type 1 diabetes, for example, is characterized by an autoimmune-mediated loss of beta cell function, but subgroups have been identified with residual beta cell function (2). Furthermore, neonatal diabetes, previously considered to be type 1 diabetes, has been reclassified as a monogenic form of diabetes, with a distinct pathophysiology and treatment pathway, enabling early administration of effective treatment (3). Studies of heterogeneity in type 2 diabetes exploring, for example, subgroups in clinical presentation and response to treatment and complication risk and ascertained using modern statistical techniques such as clustering, are increasingly prevalent in the medical literature (4, 5). In gestational diabetes mellitus (GDM), however, the potential for disease heterogeneity has only recently been considered.

GDM affects around 14% of pregnant women internationally (6) and is associated with suboptimal pregnancy outcomes, including large-for-gestational-age (LGA) infants and neonatal hypoglycemia, both directly related to maternal glycemia in pregnancy (7). The diagnosis is usually made at 28 weeks’ gestation, using an oral glucose tolerance test (OGTT), and confirmed if 1 or more glucose results exceed the diagnostic thresholds (8). This approach to diagnosis suggests homogeneity and reinforces perception of a single pathological entity, with little focus on the underlying pathological process or maternal metabolic status (9). While widely accepted that factors such as ethnicity and body mass index (BMI) can influence risk for GDM, differences in diagnostic pathways make comparison between populations globally challenging. Furthermore, the OGTT gives little nuanced data about GDM pathophysiology, which remains poorly understood.

Initial efforts to identify clinically meaningful subgroups in GDM have included the study by Powe et al in which subgroups were defined according to insulin resistance and insulin secretory dysfunction (10). The predominantly insulin resistant subgroup showed differences in baseline characteristics such as higher maternal BMI, with more LGA offspring. Classifying women according to genetic subtype (11) or disease severity (12) may also yield opportunities to apply precision medicine to disease prediction or prognostication. Furthermore, and not exclusive to genotype, we recently demonstrated that patterns of lipid disturbance in GDM contributed to neonatal size and body composition, independently of maternal glucose (13). It is unknown if different causes of GDM have an impact on offspring outcome.

One approach to precision medicine in GDM is to identify subtypes using readily available clinical parameters that are measurable and affordable in many settings worldwide. For example, it has been suggested that patterns of hyperglycemia (fasting, postload, or both) during the OGTT at diagnosis might identify subgroups of women (10) with distinct clinical outcomes (14). We are not aware of any previous study that has used the OGTT hyperglycemia profile as a tool for precision medicine in GDM in women with obesity, who in the UK now comprise 18.3% of the antenatal population (15) and among whom there is a high prevalence of GDM, together with recommendation for universal screening.

We hypothesize that different OGTT patterns of glycemia in pregnancies complicated by obesity are associated with distinct clinical and biochemical characteristics, determined by impairments of insulin secretion or insulin sensitivity. Our aim was therefore to explore this in women with obesity to identify whether patterns of glycemia represent meaningful subgroups that could be used to inform precision medicine approaches to GDM management.

## Methods

### Study Design

In order to compare characteristics among women with fasting or postload hyperglycemia, or both, we used data from the UK Better Eating and Activity Trial (UPBEAT), a randomized controlled trial of a diet and exercise intervention in pregnant women with obesity (ISRCTN 89971375; REC 09/H0802/5). The study population included 1555 pregnant women (>16 years of age; BMI ≥30 kg/m^2^) with a singleton fetus. Women were approached between 2009 and 2014 and provided written informed consent prior to study enrollment. Between 15^+0^ and 18^+6^ weeks’ gestation, women were randomly assigned to either a complex lifestyle/behavioral intervention or standard antenatal care as described in detail elsewhere (16). The intervention did not significantly reduce risk of GDM or LGA, the primary outcomes, thus allowing the data to be used as a cohort for the current analysis. All participants had an OGTT (mean 27^+6^ gestational weeks) after at least 10 hours of fasting. Participants had an extra sample collected at the fasting timepoint for research purposes. Outcome data for the study were collected as described previously (16) and neonatal measures recorded immediately after birth. LGA was defined as ≥90th-centile on customized birthweight centiles.

### Classification of Participants Into Subgroups According to Pattern of Hyperglycemia

We classified UPBEAT participants into 4 groups according to their diagnosis and pattern of hyperglycemia during the OGTT using the criteria of the International Association of the Diabetes in Pregnancy Study Groups (World Health Organization; glucose fasting ≥5.1 mmol/L; glucose at 60 minutes ≥10.0 mmol/L; glucose at 120 minutes ≥8.5 mmol/L). The 4 groups were as follows: Group 1—euglycemia; glucose concentrations below thresholds for International Association of the Diabetes in Pregnancy Study Groups-defined gestational diabetes at all timepoints; Group 2: women with isolated fasting hyperglycemia (glucose ≥5.1 mmol/L at T0; glucose below thresholds at T60 and T120); Group 3: women with isolated postload hyperglycemia (Group 3; glucose T60 ≥ 10.0 mmol/L and/or glucose T120 ≥ 8.5 mmol/L; normal fasting glucose); and Group 4: women with concomitant fasting and postload hyperglycemia (glucose above threshold at either T0 and T60; T0 and T120; or all 3 timepoints).

Participants with GDM who had persistent hyperglycemia above targets were treated with metformin and/or insulin, according to National institute of Health and Care Excellence (9) guidance.

### Clinical Biochemistry

Research blood samples were kept on ice after collection, processed within 2 hours and stored at −80°C. The blood tests were taken during the diagnostic OGTT therefore before any clinical management of GDM. Analytical methods for biochemical analyses are reported in the supplementary material (17) (available at https://www.repository.cam.ac.uk/https://doi.org/10.17863/CAM.89677). Samples were collected on ice and processed promptly for analysis of insulin. Glucose concentration was measured by local clinically accredited laboratories using either a glucose oxidase or hexokinase method. To categorize women according to insulin sensitivity and secretion, we used the homeostatic model assessment (HOMA; available at https://www.dtu.ox.ac.uk/homacalculator/) to calculate HOMA2b, a beta cell function index, and HOMA2s, an insulin sensitivity index.

### Mass Spectrometry Analysis & Data Processing for Lipidomics

To assess if different patterns of glycemia are associated with different lipid phenotypes, which in turn may influence offspring size and body composition, we analyzed blood lipid metabolites using mass spectrometry. Fasting samples of plasma were used in randomized order and prepared for mass spectrometry analysis using a high throughput direct infusion mass spectrometry (18, 19, 20). After extraction, lipids from each participant were infused into an Exactive Orbitrap (Thermo, Hemel Hampstead, UK), with a TriVersa NanoMate (Advion, Ithaca, NY, USA). The Exactive Orbitrap had a 1 Hz scan rate (mass resolution 100,000 full width at half-maximum 400 m/z). The mass spectrometer was operated in full-scan mode (*m/z* 150-1200 Da). Signals of relative abundance were obtained, using the signal intensity of each species expressed relative to the total lipid signal intensity, for each participant, per mille (‰). We used XCMS (www.bioconductor.org) and Peakpicker v 2.0 [an in-house R script (20)] to process the resulting raw mass-spectrometry data. Species were identified using lists of known species (by *m/z*) according to results in both positive ion and negative ion mode (∼8k species). We ignored signals that deviated by >9 ppm, had a signal/noise ratio of <3, or were included in <50% of samples.

### Statistical Analysis

Data on maternal clinical characteristics and conventional biochemical analyses were described with mean (SD), median [interquartile range (IQR], or n (%) as appropriate. We approached the lipidomic analysis using a sparse partial least squares discriminant analysis and Student's *t*-test following the principles of candidate biomarker discovery. We used linear regression both unadjusted and adjusted for maternal age, BMI at study enrollment, ethnicity, parity, gestational age at OGTT, trial arm (intervention/control), and neonatal sex. To address the possibility of multiple testing (430 lipid variables), we used *P* ≤ .002 as the limit of statistical significance for our lipidomics analysis, based upon a modified Bonferroni correction of 0.05/sqrt(n) (19). The limit of significance was *P* < .05 for clinical and conventional biochemical characteristics. Statistical analyses were performed on Stata software, version 16.0 (StataCorp LP, College Station, TX, USA). Missing data was not imputed.

### Sample Size

The sample size for the UPBEAT randomized controlled trial aimed to identify the effect of the lifestyle upon the risk of gestational diabetes in high-risk women and is described elsewhere (16, 21). The current study uses the UPBEAT data as a cohort. Our primary outcome was to compare HOMA2b and HOMA2s in women according to subgroups defined by glycemic patterns. We considered women with fasting hyperglycemia to be the reference group and included women with postload or mixed hyperglycemia to be the comparator groups. To account for testing these 2 groups, using alpha 2.5%, the existing sample size provides >90% power to identify a 0.65 SD difference in HOMA2b (SD 186) and HOMA2s (SD 79) between subgroups. For comparison of lipid metabolites between groups, we used alpha 1% to account for the large number of variables. The sample size provides >90% power to identify a 0.7 SD difference in lipid metabolites per group.

## Results

### Clinical Characteristics According to Pattern and Severity of Hyperglycemia

According to the criteria described here, women were subdivided by group as follows: Group 1 euglycemia, n = 626; Group 2, isolated fasting hyperglycemia n = 105; Group 3, isolated postload hyperglycemia, n = 66; Group 4, concomitant fasting and postload hyperglycemia, n = 70. Women with GDM (n = 241) were of higher age and BMI compared to euglycemic women (Table 1). In the women with GDM, those with isolated fasting hyperglycemia, isolated postload hyperglycemia, or concomitant fasting and postload hyperglycemia were statistically similar with regard to age, parity, ethnicity, neonatal sex, trial arm, birthweight centile, and abdominal circumference (with or without adjustment for gestational age at delivery). Compared to women with isolated fasting hyperglycemia, women with isolated postload hyperglycemia had a significantly lower BMI (34.8 vs 37.1 kg/m^2^ in fasting group; *P* = .005) and earlier gestational age at delivery (38.8 vs 39.3 weeks in fasting group; *P* = .020). Compared to women with isolated fasting hyperglycemia, women with concomitant fasting and postload hyperglycemia also had an earlier gestational age at delivery (38.6 vs 39.3 weeks; *P* = .001).

**Table 1.** Baseline characteristics of euglycemic women and those with gestational diabetes

	Euglycaemic women (Group 1)	Women with gestational diabetes (Groups 2-4)	Gestational diabetes subgroups		
	(n = 626)	(n = 241)	Fasting hyperglycaemia (Group 2) (n = 105)	Post-prandial hyperglycaemia (Group 3) (n = 66)	Mixed hyperglycaemia (Group 4) (n = 70)
	Mean (SD)/Median (IQR)/n (%)	Mean (SD)/Median (IQR)/n (%)	Mean (SD)/Median (IQR)/n (%)	Mean (SD)/Median (IQR)/n (%)	Mean (SD)/Median (IQR)/n (%)
Age (years)	30.3 (5.7)	31.9 (4.7)	31.6 (4.7)	32.3 (5.4) *P* = .343	32.2 (4.1) *P* = .408
BMI (kg/m^2^)	34.7 (32.6-38.2)	36.3 (33-39.8)	37.1 (33.8-40.8)	34.8 (32.2-37.9) *P* = .005	35.8 (33.8-39.9) *P* = .349
Ethnicity				*P* > .050	*P* > .050
White	438 (70.0)	144 (59.8)	58 (55.2)	42 (63.6)	44 (62.9)
Black	122 (19.5)	59 (24.5)	36 (34.3)	12 (18.2)	11 (15.7)
Asian	40 (6.4)	22 (9.1)	8 (7.6)	6 (9.1)	8 (11.4)
Other	26 (4.2)	16 (6.6)	3 (2.9)	6 (9.1)	7 (10.0)
Nulliparous	291 (46.5)	100 (41.5)	44 (41.9)	21 (31.8) *P* = .187	35 (50) *P* = .292
Trial arm (Control)	325 (51.9)	125 (51.9)	49 (46.7)	38 (57.6) *P* = .166	38 (54.3) *P* = .324
Gestational age at OGTT (weeks)	27.8 (0.7)	27.7 (1.1)	27.7 (1.6)	27.7 (0.6) *P* = 0.162	27.8 (0.6) *P* = .993
Neonatal sex (Female)	291 (46.5)	118 (50.0)	52 (49.5)	34 (51.5) *P* = .800	32 (45.7) *P* = .621
Biochemistry*					
Fasting glucose (mmol/L)	4.5 (0.3)	5.3 (0.6)	5.4 (0.4)	4.6 (0.3) *P* < .001	5.8 (0.6) *P* < .001
1 hour glucose (mmol/L)	7.2 (1.4)	10.0 (2.1)	8.3 (1.3)	10.8 (1.2) *P* < .001	11.6 (2.0) *P* < .001
2 hours glucose (mmol/L)	5.5 (1.1)	7.1 (1.8)	6.2 (1.1)	7.3 (1.6) *P* < .001	8.2 (2.1) *P* < .001
Insulin (mU/L)	17.7 (12.6-30.6)	23.0 (17.2-32.2)	23.4 (17.8-31.9)	19.5 (12.6-27.6) *P* = .074	27.4 (20.7-36.6) *P* = .049
C Peptide (ng/mL)	3.4 (2.6-5.5)	4.0 (3.2-5.4)	3.9 (3.3-5.2)	3.5 (2.5-5.5) *P* = .571	4.5 (3.7-5.5) *P* = .041
Leptin [ug/L)	62.5 (47.4-83.3)	61.8 (46.8-85.8)	64.1 (52.6-92.4)	53.2 (41.6-75.1) *P* = .003	64.5 (45.5-89.4) *P* = .554
HOMA2b (%)	221 (178-314)	197.4 (159-248)	197 (158-246)	215 (173-284) *P* = .036	189 (146-219) *P* = .826
HOMA2s	40.6 (25.4-56.3)	30.1 (22.1-39.5)	29.2 (21.7-38)	37.5 (26.9-56.7) *P* = <.001	24.8 (20.2-32.1) *P* = .207
Total chol (mmol/L)	6.0 (5.3-6.8)	6.0 (5.2-6.7)	6.0 (5.3-6.7)	6.3 (5.3-6.8) *P* = .993	5.5 (4.8-6.6) *P* = .097
LDL chol (mmol/L)	3.8 (3.1-4.5)	3.7 (2.9-4.4)	3.7 (3.0-4.4)	3.8 (2.9-4.5) *P* = .652	3.2 (2.8-3.9) *P* = .050
HDL chol (mmol/L)	1.8 (1.6-2.2)	1.8 (1.5-2.1)	1.8 (1.6-2.1)	1.7 (1.5-2.1) *P* = .346	1.7 (1.4-2.1) *P* = .230
Triglycerides (mmol/L)	1.9 (1.5-2.4)	2.1 (1.6-2.7)	2.0 (1.6-2.6)	2.1 (1.8-2.7) *P* = .076	2.2 (1.6-2.8) *P* = .022
Pregnancy outcomes					
Gestational age at birth (weeks)	40.3 (39.1-41.1)	39 (38.1-40)	39.3 (38.4-40.4)	38.8 (38.1-39.6) *P* = .020	38.6 (38-39.4) *P* = .001
Large for gestational age	44/616 (7.0)	34/241 (14.1)	11/105 (10.5)	9/66 (13.6) *P* = .532	14/70 (20.0) *P* = .082
Birthweight centile	42.1 (20.4-69.8) n = 625	53.3 (29.9-80.2) n = 240	53.4 (30.5-80.1) n = 104	49.8 (26.2-67.3) n = 66 *P* = .146	61.5 (40.7-84.2) n = 70 *P* = .262
Neonatal abdominal circumference (mm)	32.4 (30.9-34.0) n = 294	32.3 (31.1-34.1) n = 121	32.6 (31.1-34.4) n = 53	32.0 (30.4-3.7) n = 37 *P* = .127	31.8 (31.0-33.7) n = 31 *P* = .205

Abbreviations: BMI, body mass index; chol, cholesterol; HDL, high density lipoprotein; HOMA, homostatic model assessment; IQR, interquartile range; LDL, low density lipoprotein; OGTT, oral glucose tolerance test.

Data are presented as mean (SD)/median (IQR)/n (%) as appropriate. Significance testing (compared to fasting hyperglycemia group) was conducted using multinomial logistic regression.

*Analyses performed on the fasting sample at OGTT.

Missing data: Glucose at time 1 hour (n = 40), glucose at 2 hours (n = 2), gestational age at OGTT (n = 2), insulin (n = 3), C-peptide (n = 8), HOMA2b/HOMA2s (n = 19), LDL chol (n = 5), HDL chol (n = 5), triglycerides (n = 10), birthweight centile (n = 2), neonatal abdominal circumference (n = 452; missing equally across groups).

### Biochemical Characteristics According to Pattern and Severity of Hyperglycemia

Biochemical assessment showed some differences between groups; as anticipated, women with GDM had higher concentrations of glucose and insulin than euglycemic women, with evidence of insulin resistance (Table 1). Compared to women with isolated fasting hyperglycemia, women with isolated postload hyperglycemia had a higher HOMA2b score (215% vs 197%; *P* = .036) and higher HOMA2s (37.5% vs 29.2%; *P* < .001). Compared to women with isolated fasting hyperglycemia, women with concomitant fasting and postload hyperglycemia had higher concentrations of insulin (27.4 vs 23.4 mU/L; *P* = .049) and C-peptide (4.5 vs 3.9 ng/mL; *P* = .041) but no significant differences in HOMA2b or HOMA2s scores at diagnosis.

**Figure 1.**
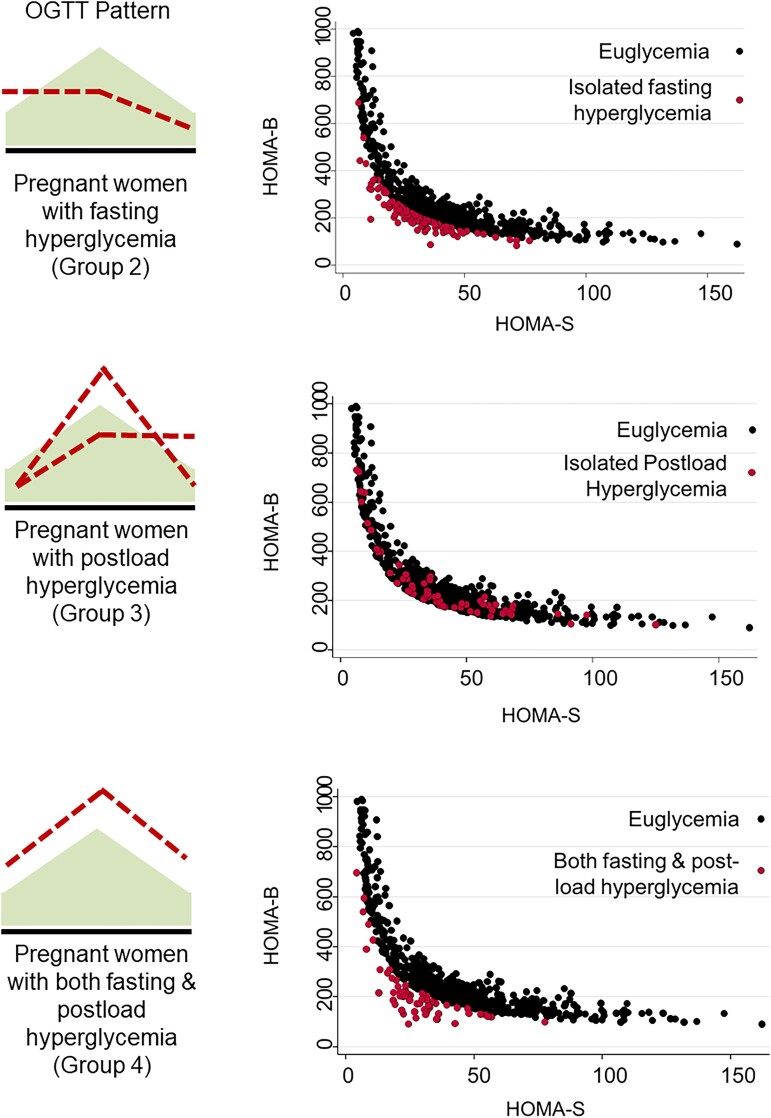
HOMA-B and HOMA-S indices in women with obesity euglycemia in pregnancy compared to gestational diabetes categorized according to patterns of glycemia.Abbreviations: HOMA, homeostatic model assessment.

Plotting insulin secretory function (HOMA2b) against insulin sensitivity (HOMA2s) in women with GDM or glycemic subgroups revealed distinct patterns (Fig. 1). As a whole group, women with GDM have low insulin secretion and insulin sensitivity. This was most evident in women with isolated fasting hyperglycemia and women with concomitant fasting and postload hyperglycemia. Unexpectedly, women with postload hyperglycemia (raised glucose at 1-hour and/or 2-hour timepoints during the OGTT) had comparable insulin indices as the euglycemic women in the cohort. Although the number of women was small, those with the most severe hyperglycemia (both fasting and postload) had evidence of the most severe defects in insulin secretion and sensitivity (Fig. 1).

Conventional clinical biochemistry analyses of lipoproteins, cholesterol, and triglycerides (TGs) did not identify any significant difference in total cholesterol between subgroups, but women with concomitant fasting and postload hyperglycemia had higher TGs (2.2 vs 2.0 mmol/L; *P* = .022) and lower LDL cholesterol (3.2 vs 3.7 mmol/L; *P* = .050) compared to women with isolated fasting hyperglycemia. Compared to women with isolated fasting hyperglycemia, women with isolated postload hyperglycemia had a lower leptin concentration (53.2 vs 64.1 ug/L; *P* = .003), consistent with their lower BMI.

### Associations Between Lipid Species and Pattern and Severity of Hyperglycemia

**Figure 2.**
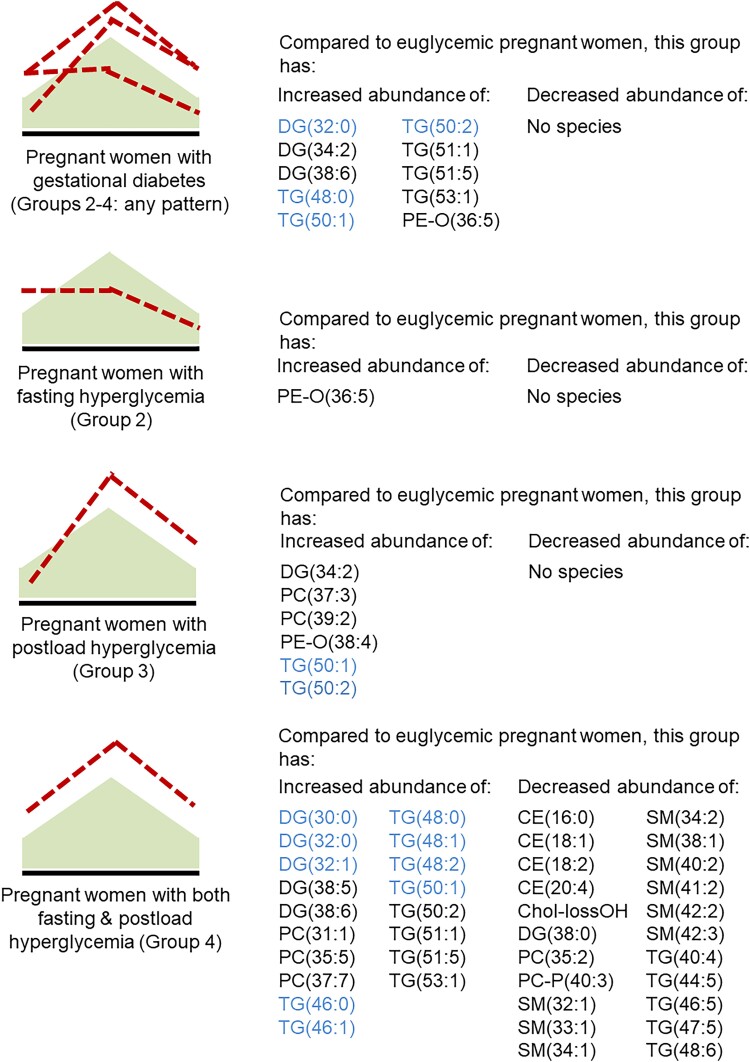
Comparison of lipidomic species abundance in maternal plasma at 28 weeks’ gestation in obese euglycemic women compared to all women with GDM (Groups 2-4), women with GDM and isolated fasting hyperglycemia (Group 2), women with GDM and postload hyperglycemia only (Group 3), and women with both fasting and postload hyperglycemia (Group 4). Only lipid variables meeting the threshold of significance (*P* ≤ 0.002) are shown and include cholesteryl esters, diglycerides, phosphatidylcholines, phosphatidylcholine-plasmalogens, phosphatidylethanolamines, phosphatidylethanolamine-plasmalogens, polyunsaturated fatty acids, sphingomyelins, and triglycerides. (Triglycerides and diglycerides related to de novo lipogenesis are shown in blue).Abbreviation: GDM, gestational diabetes mellitus.

In total, 430 lipid variables were identified including 211 in positive ionization mode and 219 in negative ionization mode (Fig. 1; supplementary material at https://www.repository.cam.ac.uk/) (17). Women with concomitant fasting and postload hyperglycemia showed the most marked lipid disturbances with significant increases in abundance of TGs associated with de novo lipogenesis and reduced abundance of cholesteryl esters (CEs), phosphatidylcholines (PCs), light TGs containing polyunsaturated fatty acids (PUFAs; ie, TGs containing less than 50 carbons and 2-6 double bonds that are likely adipose-derived) and sphingomyelins (SMs), compared to euglycemic women (Fig. 2).

By contrast, participants with isolated fasting hyperglycemia showed marginal differences from euglycemic women, with only an increase in plasmalogen PE-O(36:5) (Fig. 1). Compared to Group 1, participants with isolated postload hyperglycemia (Group 3) showed increased diglyceride DG(34:2), PC(37:3), PC(39:2), PE-O(38:4), and TGs associated with de novo lipogenesis [TG(50:1) and TG(50:2)] (Fig. 1) (22). Women with isolated fasting hyperglycemia (Group 2) had significantly more PC(35:3) and PC(37:3) compared to women with isolated postload hyperglycemia (Group 3) but had no other distinctive lipidomic features.

## Discussion

### General Statement of Findings

Categorization of pregnant women with GDM and obesity according to the pattern of hyperglycemia revealed discrete differences in clinical and biochemical characteristics and lipid abundance, with the most marked changes in insulin sensitivity and secretion identified in women with concomitant fasting and postload hyperglycemia. Unexpectedly, women with isolated postload hyperglycemia appeared to have comparable insulin secretion and sensitivity as the women without GDM. Women with the most severe GDM with concomitant fasting and postload hyperglycemia demonstrated the most marked changes in insulin secretion, sensitivity, and the plasma lipidome. Key changes to the lipid composition of serum associated with disease severity included increased PCs and increased abundance of TGs associated with de novo lipogenesis with reduced abundance of SMs, CEs, and PUFA-containing TGs. This corroborates the growing evidence base of heterogeneity in women with GDM, even in a cohort of women with obesity, which might be expected to be relatively homogeneous. However, in this study, although the pattern of glycemia was related to distinct phenotypic features, overall severity of glucose derangement appeared to be most strongly associated with insulin indices and lipid abundance.

### Strengths and Limitations of the Study

A key strength of this analysis is the inclusion of a large number of prospectively collected samples from a well-characterized multiethnic cohort, representative of typical clinical populations. Samples were taken at the time of GDM diagnosis, which removes the possibility of bias from treatment or behavior change associated with GDM management. The marked heterogeneity observed in this obese cohort also undermines the general assumption of metabolic homogeneity among obese women with GDM, based on the widely-held premise that insulin resistance is the predominant cause of GDM in this group. Due to the timings of research samples, only HOMA indices were calculable that have been shown to imperfectly reflect first-phase insulin secretion (23). Although it is noted that HOMA indices are simple and pragmatic in a clinical context, future studies should aim to improve assessment of insulin secretion through additional measures, (eg, Stumvoll index).

We included adjustments for trial arm as the intervention did not influence the development of hyperglycemia (16). The lipid composition was determined on fasting samples, and postload plasma samples may show different lipid changes, but samples for 1-hour or 2-hour timepoints were not available. For delineation of subgroups, we included all participants with postload hyperglycemia (ie, hyperglycemia at either the 1-hour or 2-hour timepoint or both during the OGTT). We cannot comment on whether women with isolated 1-hour hyperglycemia had similar lipid profiles to those with isolated 2-hour hyperglycemia as statistical power was insufficient to address this question.

### Meaning of the Study Results

#### Effect of patterns of glycemia upon insulin sensitivity and secretion

We have identified that OGTT patterns of glycemia are associated with patterns of insulin secretion/sensitivity, confirming our original hypothesis. Concomitant fasting and postload hyperglycemia were associated with the most severe defects in both insulin secretion and insulin sensitivity, followed by those with isolated fasting hyperglycemia who had less severe defects in both indices. Isolated postload hyperglycemia demonstrated similar insulin indices to euglycemic women. This unexpected finding suggests that indices of insulin secretion and sensitivity do not distinguish women with postload hyperglycemia from euglycemic women and that alternative pathological mechanisms should be considered. Alternatively, it may be that the performance of the HOMA indices during the OGTT do not adequately reflect insulin secretion and/or sensitivity (23).

A seminal review described the likely underlying mechanisms of nonpregnant individuals with impaired fasting glucose, impaired glucose tolerance, and a mixed presentation, using metabolic profiles from OGTT, intravenous glucose tolerance tests, and clamp studies; these groups might be thought comparable to Groups 2, 3, and 4, respectively. In summary, individuals with impaired fasting glucose have predominant hepatic insulin resistance, normal muscle insulin sensitivity (identified through clamp studies), and a decrease in early phase (but essentially normal late phase) insulin response (secretion) during OGTT, while those with impaired glucose tolerance have mostly muscle insulin resistance with defects in both early and late insulin secretion. Those in the mixed group have severe hepatic, muscular insulin resistance and reduction in insulin secretion (24). While the results from Groups 2 and 4 from the present study appear consistent mechanistically with nonpregnant individuals, we did not identify abnormal insulin sensitivity or secretion in Group 3 (postprandial hyperglycemia group).

In addition to the heterogeneity evidenced by patterns of glycemia and severity of hyperglycemia, we also identified heterogeneity within this cohort of women with obesity, previously considered to be a homogeneous group with insulin resistance (25). Further heterogeneity may be evident in different ethnic groups requiring further study.

### Effect of Patterns of Glycaemia Upon the Plasma Lipidome

Severity of disease as assessed by glycemic response to a glucose challenge was associated with marked differences in the abundance of lipids in the circulation whereas women with isolated fasting or isolated postload hyperglycemia exhibited modest changes. Women with concomitant fasting and postload hyperglycemia had evidence of increased species associated with de novo lipogenesis (DGs and TGs), with reduced abundance of CE, SM, and TGs containing polyunsaturated fatty acids compared to euglycemic women (Fig. 2d). In this situation, de novo lipogenesis is expected to be the route for disposal of excess glucose. Reductions in maternal CE and SM may indicate increased fetal or maternal use of substrates otherwise used for membrane expansion. PCs and SMs, common phospholipids and important membrane components that generally contain essential fatty acids and contribute to the composition and physical behavior of lipid bilayer membranes, were modified in women with GDM, especially in those with isolated postload hyperglycemia or both fasting and postload hyperglycemia (Fig. 2) (26, 27).

Our study showed elevated TGs, DGs, PCs, and sphingolipids, consistent with reports from other studies [summarized in (28)]. A study from Liu using data from the Hyperglycemia and Adverse Pregnancy Outcomes study identified that palmitoleic acid was important in mediating postload metabolism in insulin resistance (29). Our findings of increased DGs and TGs likely to contain palmitoleic acid [such as TG(48:1), (50:1) and (50:2)] are consistent with these results. Although Law and colleagues identified reductions in unsaturated phospholipids in GDM (30), conversely we found very few species were reduced in abundance in women with GDM, except in women with the most marked hyperglycemia. Women with milder forms of GDM, with hyperglycemia at one timepoint only, had less lipid disturbance. Reductions in the availability of PUFAs have previously been described in the context of diabetes in pregnancy (31-33). This could be related to maternal diet or differences in metabolism in maternal or placenta-fetal compartments. We identified only slight changes in total TG abundance with pattern of hyperglycemia, but TGs containing PUFAs were less abundant in women with concomitant fasting and postload hyperglycemia compared to euglycemic women (Fig. 2).

## Conclusions

We provide evidence of heterogeneity among women with obesity and GDM, previously considered a pathophysiologically homogeneous group, when categorized by the presence of fasting, postload, or mixed hyperglycemia. The most marked phenotypic, biochemical, and lipidomic changes were identified in women with mixed hyperglycemia. Patterns of glycemia during the OGTT may contribute to a precision approach to GDM as assessed by differences in insulin resistance/secretion. Further research is indicated to determine if isolated postload hyperglycemia reflects a different mechanistic pathway for targeted management.

## Data Availability

Data are available upon request, subject to approval from the UPBEAT consortium. Notes on analytical procedures used in this manuscript are available at https://doi.org/10.17863/CAM.89677.

## Abbreviations

CE: cholesteryl ester
DG: diglyceride
GDM: gestational diabetes mellitus
HOMA: homeostatic model assessment
OGTT: oral glucose tolerance test
PC: phosphatidylcholine
PE: phosphatidylethanolamine
PE-O: phosphatidylethanolamine plasmalogen
SM: sphingomyelin
TG: triglyceride
